# Heterotopic ossification in the abdominal wall after midline incision: a case report

**DOI:** 10.1093/jscr/rjag1000

**Published:** 2026-11-10

**Authors:** Jun Hee Han, Sung Il Kang, Sohyun Kim

**Affiliations:** Department of Surgery, Yeungnam University Medical Center, 170 Hyeonchung-ro, Nam-gu, Daegu 42415, Republic of Korea; Department of Surgery, Yeungnam University Medical Center, 170 Hyeonchung-ro, Nam-gu, Daegu 42415, Republic of Korea; Department of Surgery, Yeungnam University Medical Center, 170 Hyeonchung-ro, Nam-gu, Daegu 42415, Republic of Korea

**Keywords:** Hartmann procedure, heterotopic ossification, myositis ossificans, surgical wound

## Abstract

Heterotopic ossification (HO) of the abdominal wall following a midline incision is a rare postoperative complication characterized by mature bone formation in soft tissue. We report a 70-year-old woman who underwent emergency Hartmann procedure for perforated sigmoid diverticulitis. Four months later, preoperative computed tomography for colostomy reversal incidentally revealed two ossified lesions along the previous surgical wound: a 21-mm inverted V-shaped lesion and a 70-mm inverted J-shaped lesion. Both lesions were completely resected during open stoma reversal, and histopathological examination confirmed mature heterotopic bone. Unlike the reported male predominance, this patient was female. Although the pathogenesis remains uncertain, proposed mechanisms include periosteal implantation, mesenchymal cell differentiation induced by inflammation, and excessive suture-line tension. This case demonstrates that abdominal wall HO can develop rapidly, attain considerable size despite the absence of symptoms, and should be considered in the differential diagnosis of postoperative abdominal wall masses.

## Introduction

Heterotopic ossification (HO) refers to the formation of mature bone in tissues where bone is not normally present. Bone formation occurring within abdominal wound tissue represents a form of HO. Myositis ossificans traumatica (MOT) is characterized by ectopic bone formation following trauma or surgical intervention and has been recognized for many years. HO is a subtype of MOT [1]. Such heterotopic bone formation may cause considerable physical and psychological distress and can mimic retained foreign bodies or recurrent malignancy on clinical or radiologic assessment. Complete surgical excision with primary closure remains the standard treatment.

## Case report

In April 2022, a 70-year-old woman presented with a one-day history of sudden-onset lower abdominal pain. She had a medical history of hypertension, type 2 diabetes mellitus, and hyperlipidemia and had no prior history of abdominal surgery. Abdominal computed tomography (CT) demonstrated findings suggestive of perforated sigmoid diverticulitis. The patient subsequently underwent emergency Hartmann’s procedure. Histopathological examination confirmed a diverticulum with abscess formation. Her postoperative course was uneventful, and she was discharged on postoperative day 10.

In August 2022, ~4 months after the initial surgery, the patient was readmitted for colostomy reversal. Preoperative ACT demonstrated two well-defined hyperattenuating lesions with bone attenuation along the previous midline incision. The first lesion was an ~21-mm inverted V-shaped ossified lesion located near the superior aspect of the incision (Fig. 1). The second lesion was an ~70-mm inverted J-shaped ossified lesion involving the periumbilical region and extending toward the inferior aspect of the incision (Fig. 2).

**Figure 1:**
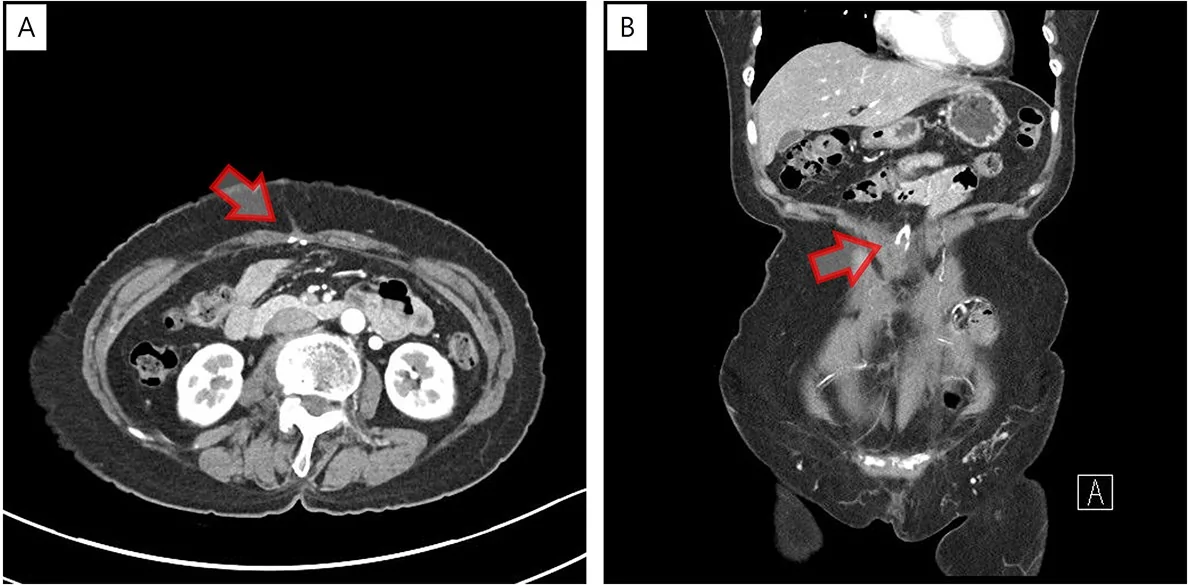
Preoperative contrast-enhanced abdominal CT obtained before stoma reversal demonstrates heterotopic ossification along the superior aspect of the previous midline incision. (A) Axial image showing an ossified lesion within the fascial layer (arrows). (B) Coronal image demonstrating an ~21-mm inverted V-shaped ossified lesion.

**Figure 2:**
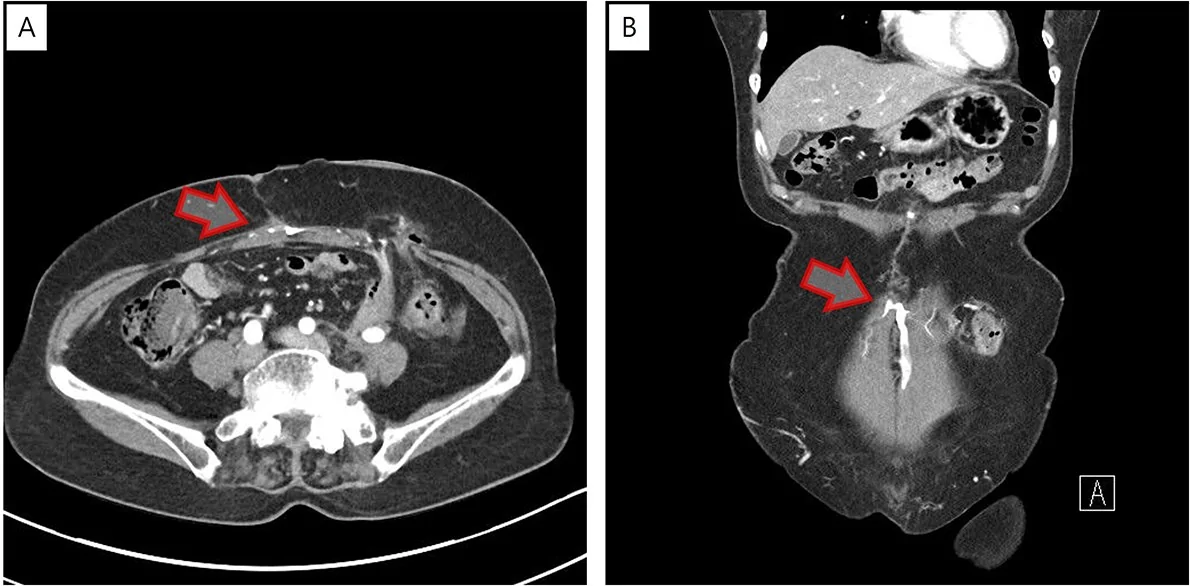
Preoperative contrast-enhanced abdominal CT obtained before stoma reversal demonstrates heterotopic ossification along the inferior aspect of the previous midline incision. (A) Axial image showing an ossified lesion located immediately superficial to the fascial layer. (B) Coronal image demonstrating an ~70-mm inverted J-shaped ossified lesion (arrows) extending longitudinally along the fascial plane.

A laparoscopic stoma reversal was initially attempted but was converted to an open procedure because of severe postoperative adhesions. The abdominal cavity was entered through the previous midline incision. Intraoperatively, two mature bony structures were grossly identified along the incision line and were completely resected (Fig. 3).

**Figure 3:**
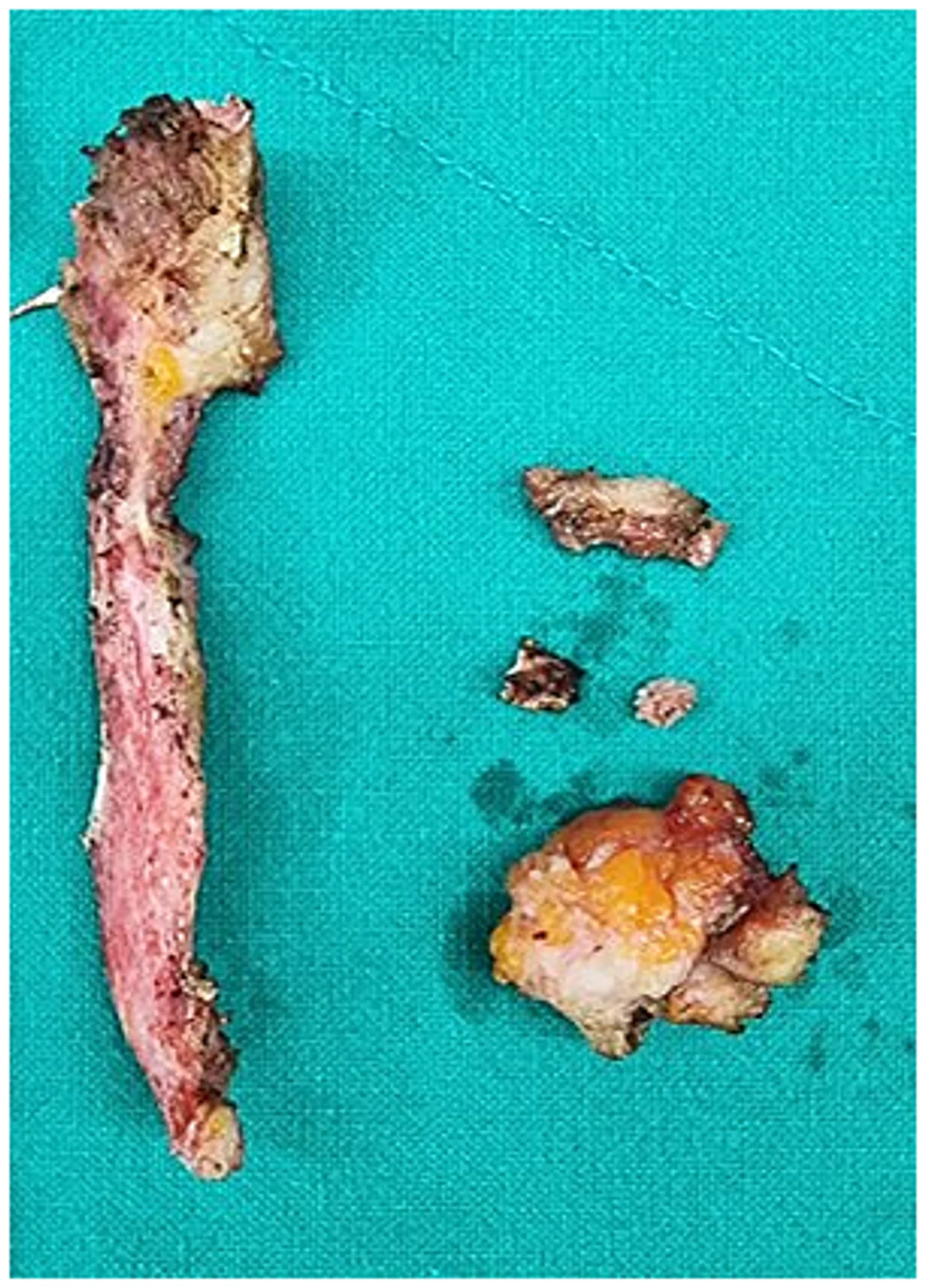
Surgically excised heterotopic ossification specimens. The left specimen represents the lesion from the inferior portion of the midline incision, and the right specimen represents the fragmented lesion from the superior portion of the midline incision.

The superior HO lesion was identified within the fascial layer, measuring 21 × 14 mm and located ~ 120 mm distal to the xiphoid process. The inferior HO lesion measured 70 × 14 mm and was situated immediately superficial to the fascial layer, with its inferior margin ~45 mm cephalad to the superior border of the pubic symphysis.

Histopathological examination of the resected specimens demonstrated mature heterotopic bone formation associated with fibrosis and fat necrosis (Fig. 4).

**Figure 4:**
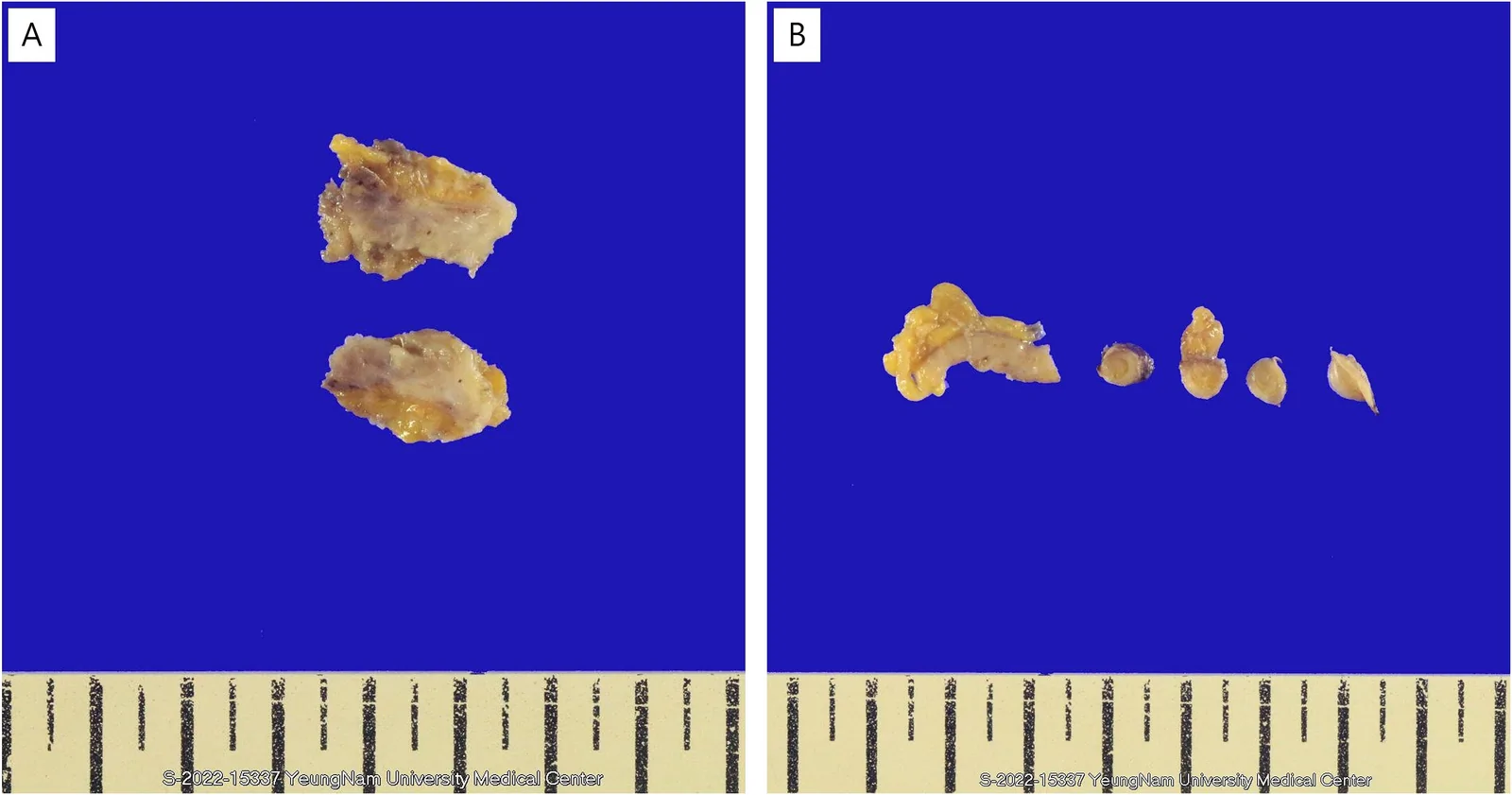
Gross pathological appearance of the ossified lesions after specimen processing. Mature heterotopic bone formation associated with fibrosis and fat necrosis is observed. (A) Lesion from the superior aspect of the midline incision. (B) Lesion from the inferior aspect of the midline incision.

The postoperative course was uneventful, and no significant abnormalities were detected during outpatient follow-up after discharge.

## Discussion

The true incidence of HO after a midline abdominal incision is unknown, and the condition is generally regarded as a rare postoperative complication. A striking male predominance has been reported, with an approximate male-to-female ratio of 10:1 [1]. Notably, our patient was female. To date, neither the reason for this sex disparity nor the underlying pathophysiological mechanism has been elucidated.

Three theories have been proposed to explain the development of HO in abdominal wounds. The first theory suggests that small periosteal fragments originating from the xiphoid process or pubic symphysis become implanted within the surgical wound and subsequently form bone [2]. In the present case, however, the midline abdominal incision did not expose either the xiphoid process or the pubic symphysis, and the considerable distance between these anatomical structures and the lesions makes this mechanism less likely. The second theory proposes that heterotopic bone formation results from immature pluripotent mesenchymal cells differentiating into osteoblasts or chondroblasts in response to local inflammatory stimuli [2]. The third theory attributes HO to excessive suture-line tension. According to this hypothesis, excessive tension may dislodge periosteal fragments from sites of muscular insertion into bone, allowing them to become implanted within the suture line and subsequently undergo ossification.

Previous studies have suggested that ectopic bone formation after a midline abdominal incision typically becomes apparent several months after surgery and is usually identified within the first postoperative year [1]. In the present case, the patient was readmitted ~4 months after the initial surgery for stoma reversal, allowing incidental identification of the lesions during preoperative evaluation. Despite the absence of symptoms attributable to HO, abdominal CT demonstrated two distinct ossified lesions measuring 21 × 14 mm and 70 × 14 mm, respectively, both of which were of considerable size.

Lateral plain radiographs are useful for identifying linear calcified structures with bone density within the abdominal wall. On ultrasonography, a hyperechoic linear structure associated with posterior acoustic shadowing can be observed. CT can also demonstrate ossified lesions along the previous surgical incision.

Although the clinical course of HO is generally benign and no direct evidence of malignant transformation has been reported, careful differentiation from other postoperative abdominal wall lesions is required. The differential diagnosis includes benign entities such as wound infection and seroma, as well as metastatic implantation of malignant cells within the abdominal wall following oncologic surgery [3].

Prophylactic short-term administration of nonsteroidal anti-inflammatory drugs (NSAIDs) has been suggested to reduce the risk of HO formation [4]. In general, asymptomatic lesions may be managed conservatively without surgical intervention, whereas symptomatic lesions are treated by complete surgical excision with primary closure [5].

Future investigations are needed to better understand the pathogenesis of HO, including its relationship with underlying patient comorbidities and the characteristics of susceptible patient populations. In addition, studies using serial lateral plain radiographs may help clarify the temporal development of HO. Further research is also required to establish effective preventive strategies, including optimization of suture-line tension and prophylactic measures beyond the use of NSAIDs.
